# Genome-wide association study of the age of onset of type 1 diabetes reveals HTATIP2 as a novel T cell regulator

**DOI:** 10.3389/fimmu.2023.1101488

**Published:** 2023-02-01

**Authors:** Christopher J. Cardinale, Xiao Chang, Zhi Wei, Hui-Qi Qu, Jonathan P. Bradfield, Constantin Polychronakos, Hakon Hakonarson

**Affiliations:** ^1^ Center for Applied Genomics, Children’s Hospital of Philadelphia, Philadelphia, PA, United States; ^2^ College of Artificial Intelligence and Big Data For Medical Sciences, Shandong First Medical University & Shandong Academy of Medical Sciences, Shandong, China; ^3^ Department of Computer Science, New Jersey Institute of Technology, Newark, NJ, United States; ^4^ Quantinuum Research LLC, Wayne, PA, United States; ^5^ Department of Pediatrics, McGill University, Montreal, QC, Canada; ^6^ Department of Pediatrics, Perelman School of Medicine at the University of Pennsylvania, Philadelphia, PA, United States

**Keywords:** genome-wide association study (GWAS), T cells, RNA-seq, pediatrics, gene expression, HTATIP2/TIP30, type 1 diabetes

## Abstract

**Introduction:**

Type 1 diabetes, a disorder caused by autoimmune destruction of pancreatic insulin-producing cells, is more difficult to manage when it presents at a younger age. We sought to identify genetic correlates of the age of onset by conducting the first genome-wide association study (GWAS) treating the age of first diagnosis as a quantitative trait.

**Methods:**

We performed GWAS with a discovery cohort of 4,014 cases and a replication cohort of 493 independent cases. Genome-wide significant SNPs were mapped to a causal variant by Bayesian conditional analysis and gel shift assay. The causal protein-coding gene was identified and characterized by RNA interference treatment of primary human pan-CD4+ T cells with RNA-seq of the transcriptome. The candidate gene was evaluated functionally in primary cells by CD69 staining and proliferation assays.

**Results:**

Our GWAS replicated the known association of the age of diagnosis with the human leukocyte antigen complex (HLA-DQB1). The second signal identified was in an intron of the NELL1 gene on chromosome 11 and fine-mapped to variant rs10833518 (P < 1.54 × 10^−9^). Homozygosity for the risk allele leads to average age of onset one year earlier. Knock-down of HIV TAT-interacting protein 2 (HTATIP2), but not other genes in the locus, resulted in alterations to gene expression in signal transduction pathways including MAP kinases and PI3-kinase. Higher levels of HTATIP2 expression are associated with increased viability, proliferation, and activation of T cells in the presence of signals from antigen and cytokine receptors.

**Discussion:**

This study implicates HTATIP2 as a new type 1 diabetes gene acting via T cell regulation. Larger population sample sizes are expected to reveal additional loci.

## 1 Introduction

Type 1 diabetes (T1D) is a disorder in which the immune system destroys the insulin-producing β cells of the pancreatic islets, resulting in a potentially fatal inability to utilize glucose and numerous co-morbidities from chronic hyperglycemia. The liability to develop the disorder is one of the most heritable of polygenic traits, with much of the genetic risk originating in the major histocompatibility complex (*MHC*) locus on chromosome 6, containing the human leukocyte antigen (HLA) genes (1).

The age-of-onset of T1D is bimodal, with peaks at ages four (females) and ten (males) (2). An earlier presentation of disease is clinically concerning because it is more difficult to manage in younger patients. Accordingly, genetic studies have been pursued to identify risk factors for an earlier age of onset, identifying the HLA class II DRB1-DQB1 locus and several class I alleles (3, 4). This result is not unexpected considering the MHC’s predominant role (about 50% of heritability for T1D) in the risk for developing disease compared to the general population (5). However, since the MHC explains only a few percent of variance in the age of onset, other loci are involved through a complex mode of inheritance.

Genome-wide association studies (GWAS) in T1D have been a powerful tool to identify single nucleotide polymorphisms (SNPs) that predispose to a phenotype, even with weak effects (6–8). The most recent GWAS using a case/control approach found 92 loci with genome-wide significance (*P* < 5 × 10^−8^) (9). Data on the age-of-onset is not available in most public genotype datasets, however, there are some cohorts that include this information. In this study, we applied GWAS to T1D cases with available age-of-onset data in discovery and replication cohorts in order to discover SNPs associated with the subjects’ age at initial diagnosis. Since the age at diagnosis is less heritable than the development of disease regardless of age, our discovery cohort of 4,014 cases only afforded sufficient power to detect two associations. However, since one of the two signals is novel, and not identified in case/control GWAS, this study nominates a new gene for mechanistic study that will provide insight in T cell regulation and type 1 diabetes susceptibility.

## 2 Materials and methods

### 2.1 Genome-wide association study

In the discovery set, whole-genome genotype data and age-of-onset information for 4,129 T1D patients were downloaded from dbGAP (accession: phs000180.v3.p2) provided by the Genotype Type 1 Diabetes Genetics Consortium (T1DGC) (10). All samples were genotyped on the HumanHap550 SNP array (Illumina). Inclusion criteria of T1D patients were i) type 1 diabetes diagnosis, ii) insulin dependent for at least 6 months, iii) diagnosed between the ages of 6 months and 16 years inclusive (11).

The replication cohort and accompanying data were collected in pediatric diabetes clinics in Montreal, Toronto, Ottawa, Winnipeg and the Children’s Hospital of Philadelphia. All samples were genotyped with the HumanHap550 SNP array. These subjects have been treated with insulin since diagnosis and none has stopped treatment for any reason since (7).

SNPs with genotype missing rate > 5%, minor allele frequency < 1%, and Hardy-Weinberg equilibrium *P* value < 10^−5^ were excluded. After quality control, 533,856 and 525,261 SNPs were kept in the discovery set and replication set respectively for the imputation analysis. To remove cryptic relatedness between samples, we calculated the identity-by-descent (IBD) scores and remove one individual in the pairs of subjects with IBD > 0.25. We conducted principal components analysis among three cohorts separately using EIGENSTRAT to detect and correct for potential substructures and outliers (**Supplementary Figure S1**) (12). A total of 4,014 and 493 subjects were kept in the discovery set and replication set, respectively, for the imputation analysis and association test.

Genotype imputation was conducted with IMPUTE2 using the reference panel 1000 Genome Phase I integrated variants set December 2013 release (13). We used SHAPEIT recommended by Howie et al. to infer the haplotypes before imputation (14). After imputation, 6,988,016 and 6,636,371 SNPs were kept for the association test. In consideration of the uncertainty of imputation, association analysis of the imputed genotypes was calculated with the SNPTEST v2 package (15) with sex and the first ten principal components of the population substructure analysis as covariates. Meta-analysis was performed by PLINK (16). Fixed effects *P* values were reported.

SNPTEST was used to calculate Bayes factors for each SNP in the chromosome 11 locus using age of onset as a quantitative trait. The credible set analysis was then performed with the Python script credible_set_analysis.py, which can be found at https://github.com/edm1/Credible-set-analysis/blob/master/credible_set_analysis.py. The posterior probabilities were summed from a list sorted by Bayes factors until the cumulative sum was equal to or greater than 0.95. This new subset of SNPs is taken to be the 95% credible set.

### 2.2 Electrophoretic mobility shift assay

Probes for gel shift were synthesized as complementary pairs of oligonucleotides by Integrated DNA Technologies (Coralville, IA). The 35-bp oligos were centered on the SNPs of interest and a separate probe was made for each allele. 1.5 pmol of the oligos were annealed in polynucleotide kinase buffer (New England Biolabs, Ipswich, MA). To this solution was added 10 units T4 PNK and 30 µCi of ^32^P-[γ]-ATP (Perkin Elmer, Waltham, MA) at 37°C for 30 min. The PNK was removed by two extractions with StrataClean resin (Agilent Technologies, Santa Clara, CA) and the probes were desalted with ProbeQuant Sephadex columns (Cytiva, Marlborough, MA).

Nuclear extract of U-937 histiocytic lymphoma cells was purchased from Active Motif (Carlsbad, CA). Each binding reaction was 30 µl in volume, and was composed of 20 mM Tris pH 7.5, 5 mM MgCl_2_, 2.5% glycerol, 0.1 mg/ml poly-dIdC, 50 fmol labeled probe, 2.5 pmol competitor (where applicable), and 2 µl of nuclear extract. Binding took place at room temperature for 30 min. To this reaction was added 6× loading buffer (40% sucrose). A large-format 4% polyacrylamide gel with 0.5× TBE buffer was pre-electrophoresed at 200 V for 2 h. The samples, including a probe-only control, were loaded and electrophoresed at 200 V for 2.5 h. The gel was exposed to a phosphorimager screen (Cytiva) overnight. The screen was scanned on a Typhoon 9500 imager (Cytiva) and analyzed in ImageJ.

### 2.3 T cell culture and transfection

Leukapheresis was performed on healthy human donors by the University of Pennsylvania Human Immunology Core facility followed by isolation of the CD4^+^ T cells by RosetteSep (STEMCELL Technologies, Vancouver, BC) by the facility. The donors are of equally male and female sexes with age range 21 to 60. The core facility operates under its own IRB protocol and our use of these cells is considered secondary use of a de-identified biospecimen by our IRB.

Fifteen million freshly isolated cells were cultured in 30 ml RPMI-1640 with 10% fetal bovine serum at 37°C and 5% CO_2_. No antibiotics were used. Cell growth took place in 5 ml of culture per well in a 6-well plate in the presence of 10^6^ beads/ml of Dynabeads Human T-Activator CD3/CD28 (Thermo Fisher Scientific, Waltham, MA). The cells were cultured for 48-72 h prior to transfection in the presence of beads, resulting in one to two doublings.

Transfections used the Neon Transfection System electroporator (Thermo Fisher Scientific) and the 100-µl kit after 48-72 h expansion in culture. The cell suspensions were sheared from the beads by pipetting up and down, and the beads were removed with a magnet. Three million cells per transfection were centrifuged and washed in 5 ml PBS. The cell pellet was resuspended at 20 million cells/ml in Neon Buffer T. Each electroporation received 2 million cells and 100 pmol siRNA or 10 µg mRNA in a 100 µl volume. The electroporator settings were 1700 V, 2 pulses, 15 msec. The transfected cell suspensions were immediately transferred to 2 or 3 ml of warm RPMI/10% FBS and allowed to recover in the incubator for 48-72 h. T cell receptor stimulation took place after this recovery period. In our testing with a green fluorescent protein-encoding plasmid, we observed 70% transfection efficiency and 50% viability.

### 2.4 Transcriptome analysis

Primary CD4^+^ T cells of a 42-year-old male donor were expanded in culture by activation with Dynabeads CD3/CD28 and 10 ng/ml IL-2 for 72 h. The cells were electroporated in duplicate with siRNA SMARTpools against HTATIP2, PRMT3, FANCF, SVIP, and non-targeting control #3 (Horizon Discovery/Dharmacon, Cambridge, UK). siRNA sequences are in the **Online Supplement**. The transfected cultures incubated for 72 h. RNA was extracted with an RNeasy mini kit (QIAGEN, Düsseldorf, Germany) including on-column DNase digestion according to the manufacturer’s protocol. Quality control was performed by NanoDrop spectrophotometry and TapeStation 4400 (Agilent Technologies, Santa Clara, CA) to verify an RNA Integrity Number > 9.

RNA-seq was performed using the classic TruSeq Stranded mRNA kit (Illumina, San Diego, CA) adapted to automated processing on the Sciclone liquid handling robot (Perkin Elmer). Each quality-controlled library was sequenced on a Novaseq 6000 (Illumina) using V1.5 chemistry, in paired-end mode with a read length of 2 × 100 bp reaching 40 × 10^6^ reads per sample.

The paired-end output was demultiplexed to generate FASTQ files and then assembled to the hg19 (1K Genomes Project) reference genome to generate BAM files using the DRAGEN hardware and software pipeline for RNA-seq (Illumina). The table of read counts per million was produced from the BAM files using featureCounts (17) to assign reads to the Gencode GFF3 annotations. The resulting expression matrix was uploaded to WebMeV (18) to perform differential gene expression analysis using edgeR (19) with TMM normalization. Principal components analysis was performed on the normalized counts-per-million (CPM) table with Prism 9 (GraphPad Software, San Diego, CA). Pathway analyses were performed with Fast Gene Set Enrichment Analysis (20), DAVID (21), and Ingenuity Pathways Analysis (QIAGEN).

### 2.5 Reverse transcription-quantitative polymerase chain reaction

RNA was extracted from primary human T cells 72 h post-transfection with siRNA of HTATIP2 or a non-targeting control siRNA as described above. No stimulation was performed post-transfection. One microgram of RNA was reverse-transcribed to first-strand cDNA with the Transcriptor Universal cDNA Master kit (Roche Molecular Systems, Branchburg, NJ) at 55°C for 10 min. qPCR used the Forget-Me-Not EvaGreen kit (Biotium, Fremont, CA) in a ViiA 7 real-time PCR instrument (Thermo Fisher Scientific). The PCR reaction was performed for 40 cycles in 10 µl volume with 5 sec denaturation at 95°C and 20 sec elongation/data acquisition at 60°C. Primers to the transcripts of interest were designed in SeqBuilder (DNASTAR, Madison, WI) to span an intron > 3 kb in length to prevent amplification of genomic DNA (sequences included in **Supplement**). The experiment was repeated four times with different human donors. Housekeeping genes ALAS1 and HMBS were used to determine a ΔCt value for each transcript of interest. The ΔΔCt was calculated from the difference between the non-targeting control sample and the HTATIP2 knock-down sample and converted to a relative expression level where the non-targeting control is 1.0. The four experiments of each PCR target were compared to the 1.0 level using a one-sample *t* test in Prism 9.

### 2.6 Flow cytometry for CD69 expression

CD4^+^ T cells were isolated from a different human donor in each of four experiments. After 72 h expansion, the cells were transfected with an siRNA SMARTpool against HTATIP2 or a non-targeting control siRNA. At 72 h post-transfection, the cultures were counted, then seeded in a 24-well plate at 500,000 cells/ml. ImmunoCult Human CD3/CD28 T Cell Activator complexes (STEMCELL Technologies) were added to the wells at concentrations of 1:100, 1:200, 1:400, 1:800, 1:1600, and non-stimulated. After 24 h of activation, cells were processed for FACS analysis. Briefly, each sample was washed in PBS and stained for viability with LIVE/DEAD AlexaFluor 647 fixable viability stain (Thermo Fisher Scientific) for 30 min on ice. The cells were washed again and stained in BSA/PBS buffer with mouse IgG1κ-FITC isotype control or anti-human CD69-FITC antibodies (both from eBioscience/Thermo Fisher Scientific), or unstained, for 30 min on ice. The cells were washed with PBS and fixed with 4% formaldehyde in PBS followed by washes with BSA/PBS buffer. The cells were analyzed on the CytoFLEX 3-laser FACS analyzer (Beckman Coulter, Pasadena, CA) using 488-nm excitation for FITC and 638-nm excitation for LIVE/DEAD. The events were gated using SSC-A/SSC-H to select singlets, and then gated on low-LIVE/DEAD stain to select live cells. The threshold for CD69 positivity was set using the unstained and isotype control samples. Analysis was performed in FlowJo 10.8 (BD Bioscience, Ashland, OR).

### 2.7 Viability experiment

The control *E. coli* β-lactamase gene does not have catalytic activity in cells without its antibiotic substrate. The HTATIP2 gene was amplified from a Mammalian Gene Collection cDNA clone 3455757 (Horizon Discovery/Dharmacon). The PCR amplicons of these genes were ligated to the *Eco*RI and *Xba*I sites of pTNT (Promega, Madison, WI), a vector containing a T7 RNA polymerase promoter for *in vitro* transcription/translation. The capped-and-tailed mRNA was synthesized *in vitro* using the HiScribe T7 ARCA mRNA Kit with tailing (New England Biolabs) according to the manufacturer’s protocol. The reaction was purified by precipitation with lithium chloride. The product was quality-controlled by NanoDrop spectrophotometry and by electrophoresis on a denaturing formaldehyde/MOPS agarose gel.

Primary human CD4^+^ cells from a donor were expanded for 72 h using anti-CD3/CD28 beads and the supernatant medium from the culture was saved and filtered through a 0.22-micron sterile filter. The cells were transfected with 10 µg mRNA as described above in four replicates. The cells from electroporation were diluted to 200,000 cells/ml in fresh RPMI/FBS or 50% fresh medium with 50% filtered spent medium. The cell suspensions were seeded at 0.1 ml/well in an opaque white 96-well plate and allowed to incubate for 72 h. At that point, an equal volume of CellTiter-Glo 2.0 assay reagent (Promega) was added to each well. The luminescence was read on a SpectraMax L microplate luminometer (Molecular Devices, San Jose, CA) to measure the adenosine triphosphate (ATP) concentration as a marker of cell viability and cell number.

## 3 Results

For the discovery stage of GWAS, data were obtained from the Database of Genotypes and Phenotypes (dbGaP) from a published meta-analysis of the Type 1 Diabetes Genetics Consortium (11). Inclusions in the study were type 1 diabetes subjects with known age of onset, insulin-dependent for at least six months, and diagnosed between the ages of 6 and 16 years old. We identified and included 4,014 unrelated European patients in the GWAS after population-structure principal components and quality control analyses. Associations between genotypes and ages of onset were evaluated using a linear regression model with sex and the top ten whole-genome principal components as independent variables. The replication analysis used 493 unrelated European patients from the dataset of the Children’s Hospital of Philadelphia and McGill University (7).

The discovery analysis of directly-genotyped SNPs identified two loci associated with the age of onset of diabetes. The stronger of the two signals is in the MHC locus on chromosome 6 in the vicinity of HLA-DQB1 (rs9357152, *P* < 1.42 × 10^−8^). The second in the *NELL1* gene (rs10833518, *P* < 5.84 × 10^−8^) on chromosome 11 (**Supplementary Figure S2**). An interaction analysis between rs9357152 and rs10833518 indicated that these loci may contribute independently to the age of onset of diabetes (*P* = 0.44). Whole-genome imputation further confirmed the association of the MHC (rs114904770, *P*_imputed_ < 8.39 × 10^−12^) and *NELL1* (rs7104612, *P*_imputed_ < 4.16 × 10^−8^) loci, however, no additional loci were identified. The latter association is novel, and this locus does not appear in case/control GWAS (9).

**Figure 1:**
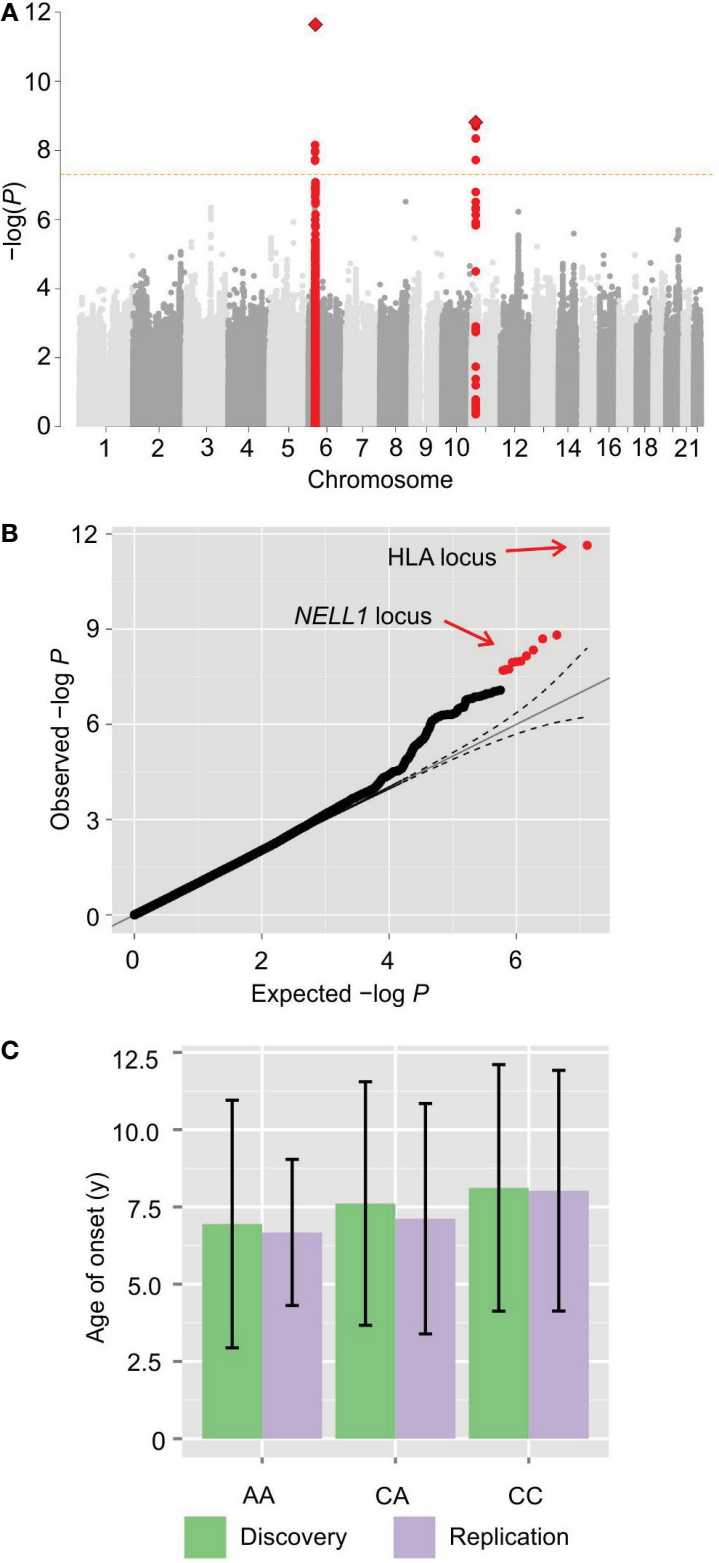
GWAS of type 1 diabetes age-of-onset reveals a novel locus. **(A)** Manhattan plot of genome-wide association statistics. The x-axis displays the autosomal genome coordinate from chromosome 1 to 22. The y-axis is the negative logarithm of the *P* value for association of each SNP with the age of onset in meta-analysis. The signal on chromosome 6 originates from the human leukocyte antigen locus. A novel signal in an intron of the NELL1 gene on chromosome 11 reaches genome-wide significance. **(B)** Q-Q plot of association *P* values. **(C)** Bar plot with mean and standard deviation of the age of onset for each genotype of rs10833518 in meta-analysis, showing a statistically-significant average age of diagnosis of one year earlier for homozygotes (*P* = 1.54 × 10^−9^, Wald test).

GWAS analysis of the replication cohort also confirmed the association between the *NELL1* locus and age of onset (rs10833518, *P*_genotyped_ < 7.34 × 10^−3^; rs7104612, *P*_imputed_ < 7.80 × 10^−3^). Meta-analysis of the discovery and replication studies indicate that the association at the *NELL1* locus surpasses genome-wide significance (rs7104612, *P*_meta_ = 1.54 × 10^−9^) (**Figure 1A**).

Examination of the quantile-quantile plot shows that the association statistics for these two loci are markedly higher than the sub-genome-wide significant SNPs (**Figure 1B**), indicating a relatively strong contribution to the heritability. Notably, there are numerous SNPs with observed *P* values that exceed the expected *P* values under the null distribution, albeit not reaching genome-wide significance, demonstrating that there is heritability for the age of onset that is not captured by the two pinpointed loci.

Regarding the lead SNP, rs10833518, homozygotes for the A (risk) allele have an average age at diagnosis one year earlier than homozygotes for the non-risk allele (*P* = 1.54 × 10^−9^ by linear regression with Wald test) (**Figure 1C**). The one-year difference implies that the locus has clinically significant impact in at least some individuals. The age of onset did not differ significantly between sexes (**Supplementary Figure S3**). The inclusion of sex as an independent variable in the regression analysis corrects for this dimorphism when calculating genotype *P* values.

### 3.1 Nomination of the causal SNP

In some GWAS loci, the strength of the association, the pattern of linkage disequilibrium, and the availability of dense and imputed genotype data make it possible to fine-map trait associations to a small number of SNPs with a total posterior probability of causality >95%, called a credible set (22). Using Bayesian conditional probability under the assumption of a single causal variant, we identified a credible set of four SNPs for the *NELL1* locus, with rs10833518 yielding a posterior probability of ~80% (**Table 1**).

**Table 1:** Fine-mapping by Bayesian conditional probability of the genetic association signal in the chromosome 11 locus.

rsID	SNP (hg38)	log BF	PP	Cumulative PP
rs10833518	chr11:21363461:C:A	5.55	0.805	0.805
rs1945454	chr11:21369355:A:C	4.52	0.075	0.880
rs7104612	chr11:21363155:A:G	4.40	0.056	0.936
rs10833519	chr11:21372139:C:T	3.78	0.014	0.950
rs12291762	chr11:21374549:C:G	3.55	0.008	0.958

The posterior probability (PP) of being the causal variant for the association is shown for each SNP. BF, Bayes’ Factor.

**Figure 2:**
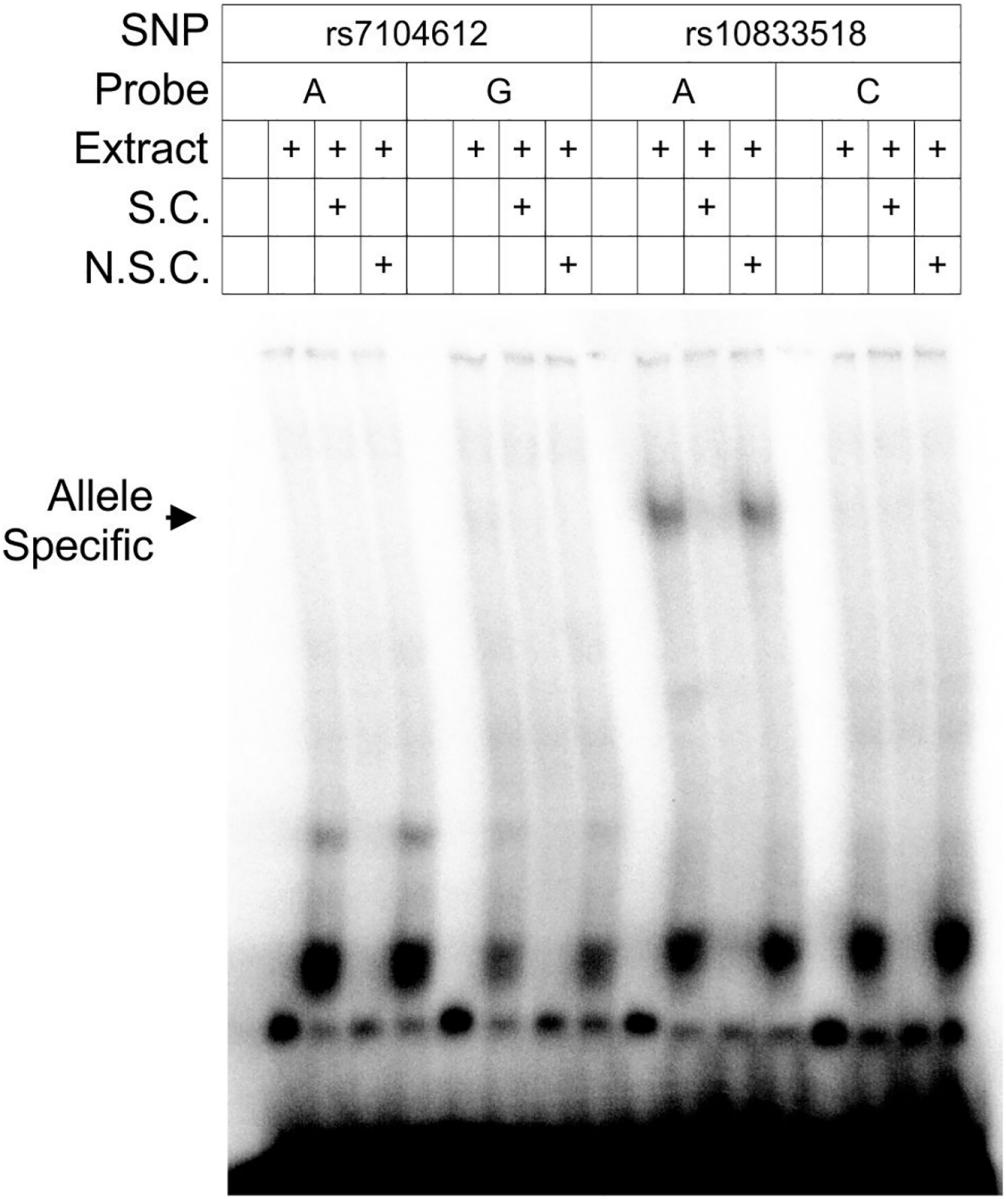
Gel shift assay demonstrates a protein-nucleic acid complex specific to the risk allele of rs10833518. Nuclear extract was obtained from U-937 histiocytic lymphoma cells and complexed with 35-bp radiolabeled probes for both alleles of the highest-association risk SNPs, rs7104612 and rs10833518. S.C., specific competitor, an unlabeled probe of the same sequence at 50× concentration. N.S.C., non-specific competitor, an unlabeled probe of scrambled sequence at 50× concentration.

We sought further evidence that this SNP may have regulatory function by an electrophoretic mobility shift assay (EMSA). Gel shift has been used previously to provide evidence that a SNP is the causal variant in GWAS associations (23). Thirty-five base pair probes centered on SNPs with the two lowest *P*-values (rs7104612 and rs10833518) were bound to nuclear extract from U-937 histiocytic lymphoma cells. The lower posterior probability SNP, rs7104612, did not show a shifted DNA-protein complex for either allele. Only the risk allele (A) of the higher posterior probability SNP, rs10833518, bound a high-molecular weight protein complex (**Figure 2**). The surrounding sequence that contains rs10833518 does not match a known binding motif for a transcription factor, therefore we cannot ascertain the identity of the nuclear proteins making up the complex. However, the Bayesian calculation and EMSA suggest that rs10833518 has a regulatory function.

### 3.2 Identification of gene candidates

The association haplotype on chromosome 11 is located within an intron of the *NELL1* gene ~700 kb from the gene’s promoter. The next-closest promoter is *ANO5*, ~800 kb away. In the event that the casual SNP influences expression of a protein-coding gene, it seems that it mediates a long-range interaction. We attempted to identify a causal gene by querying rs10833518 and rs7104612 against expression-quantitative trait locus (eQTL) databases, the GTEx Portal and eQTLgen (24), but did not find any *cis* or *trans* associations. However, it is common not to observe eQTLs due to the limited sample size of GTEx, especially in view of the multiplicity of tissues and genes, and the lack of *trans*-eQTLs in the larger eQTLgen project.

Statistical methods have been used to partition heritability for traits into different tissues or cell-types based on DNA elements (promoters, enhancers, exons, etc.) where the trait-associated SNPs reside using the linkage disequilibrium score regression technique. These analyses show that nearly all the common SNP-heritability of T1D is explained by variants located within DNA elements that are active in the immune system with few exceptions, such as the insulin gene (25–27). The lead SNPs of the *NELL1* association do not reside within an element marked by DNase hypersensitivity according to the master list of 125 cell types in the ENCODE Portal (28). However, well-validated causal SNPs have been found in inflammatory bowel disease that are not located in elements with epigenetic signatures of chromatin accessibility or histone modification (29, 30).

**Figure 3:**
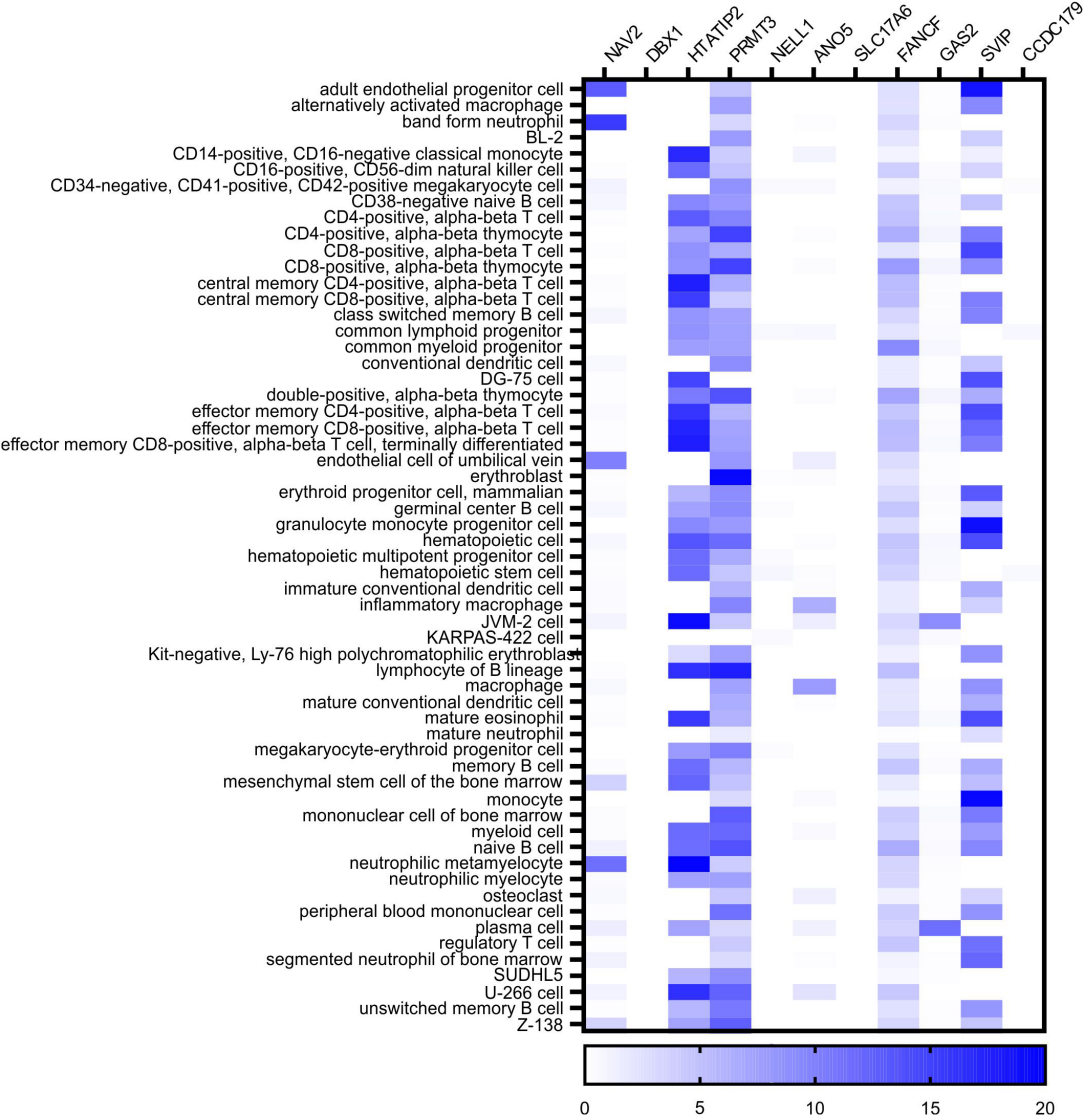
BLUEPRINT Epigenome RNA-seq data identifies four genes expressed in blood cells in the chromosome 11 locus. The count of reads in transcripts per million (TMP) was queried for each of the indicated cell types from the data of the BLUEPRINT Epigenome Consortium. The eleven protein-coding genes located in a 3-megabase window centered on the rs10833518 SNP are indicated with heatmap intensity of expression in each cell type.

In a 3-Mb window centered on the association haplotype, there are 11 protein-coding genes: *NAV2*, *DBX1*, *HTATIP2*, *PRMT3*, *NELL1*, *ANO5*, *SLC17A6*, *FANCF*, *GAS2*, *SVIP*, and *CCDC179* (**Supplementary Figure S4**). Based on information from the Protein Atlas (31, 32) and the BLUEPRINT Consortium (33) we determined that four genes (*HTATIP2*, *PRMT3*, *FANCF*, and *SVIP*) are expressed in cells of the immune system or blood (**Figure 3**). *NELL1* itself is not a compelling candidate because it is expressed mainly in the central nervous system and is absent from blood or human pancreas cells. Therefore, we remained agnostic as to the mechanism of gene regulation by rs10833518 and instead pursued a hypothesis-driven approach of testing candidate genes in the most T1D-associated cell type, T cells.

### 3.3 Transcriptomics prioritizes HIV TAT-interacting protein 2

**Figure 4:**
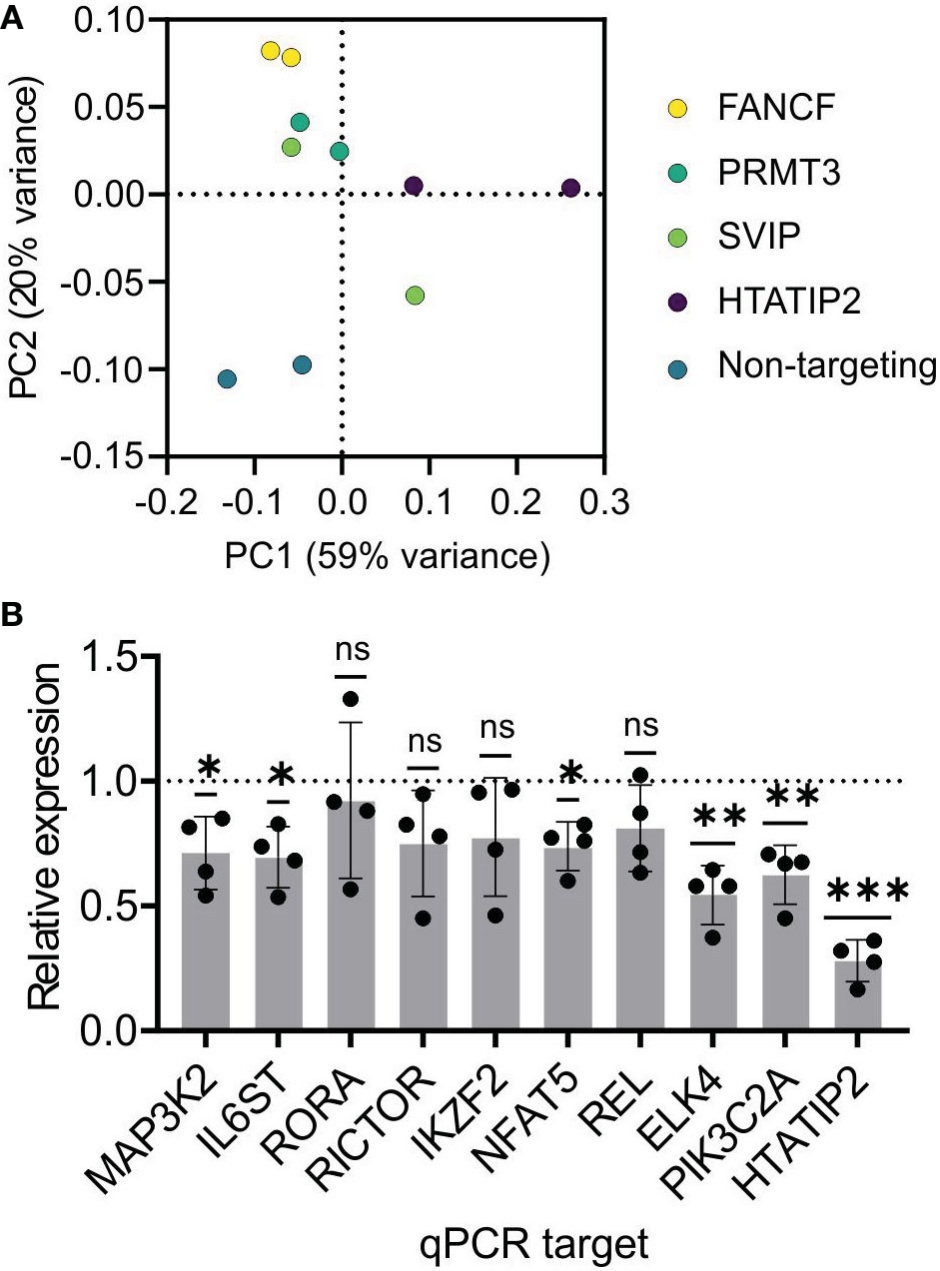
Gene expression analysis of candidate gene knock-down implicates HTATIP2 as a T cell regulator. Primary CD4^+^ T cells were electroporated with siRNA pools for the genes FANCF, PRMT3, SVIP, HTATIP2, or a non-targeting control siRNA in biological duplicates. Poly-A-tailed mRNA was subjected to transcriptome sequencing. **(A)** Principal components analysis of the ten samples color-coded by the siRNA. **(B)** Knock-down of HTATIP2 using siRNA in primary CD4^+^ T cells from four donors. mRNA levels of the indicated genes were assayed by RT-qPCR and normalized to the mRNA level of the non-targeting siRNA-transfected cells for each donor. ns, non-significant; *P < 0.05, **P < 0.01, ***P < 0.001.

We knocked-down mRNA of the four immune-expressed genes in primary human pan-CD4^+^ T cells to assess the impact on the transcriptome using RNA-seq. The cells of a healthy adult male donor were electroporated in duplicate with siRNA SMARTpools against these four genes and a non-targeting control siRNA. Poly-A^+^ mRNA was subjected to deep sequencing. Principal components analysis shows that 79% of the variance among the ten transcriptomes is explained by two dimensions. The PC1 axis explains 59% of variance and differentiates all other samples from HTATIP2 siRNA treatment. SVIP sample 1 clustered with FANCF and PRMT3, while SVIP sample 2 was an outlier. The PC2 axis explains 20% of variance and differentiates non-targeting siRNA samples from active RNA interference samples (**Figure 4A**). We confirmed the efficacy of the knock-down of the target genes themselves by the RNA-seq data.

Differential gene expression (DE) analyses were carried out using edgeR, taking each one of the four siRNA treatments in turn as the experimental group and the other eight samples as the control group. For SVIP and PRMT3 knockdowns, the only significant genes at an adjusted *P* < 0.05 were SVIP and PRMT3 themselves, arguing that they have little role in gene regulation in T cells. For the FANCF knock-down, the significant genes were GFPT2, DYM, ERCC3, PLXNA1, PLXNB2, CYBRD1, UBE2Z, FOXP4, and FANCF.

HTATIP2 knock-down, on the other hand, revealed 106 differentially expressed genes with average fold-change > 1.7. Gene Set Enrichment Analysis and DAVID analysis found that these DE genes were highly enriched in signaling pathways, including kinases, receptors, and transcription factors. Among genes of biological interest were sterile-20 kinase family member TAOK1; MAP kinase pathway members MAP3K2 and ELK4; calcineurin-NFAT member NFAT5; PI3-kinase member PIK3C2A; NF-κB member RELA; and transcription factors Helios (IKZF2) and RORA. Receptors include integrins (ITGA2 and ITGAX), IL6ST, Natural Killer Triggering Receptor (NKTR), and IgE antibody Fc receptor (FCER1G).

A selection of these DE genes based on diverse pathways were tested by siRNA to HTATIP2 using RT-qPCR and we found that all nine genes had reduced expression on average (**Figure 4B**). Five were statistically significant (*P* < 0.05 by one-sample *t* test), but we could not reject the null hypothesis for four genes either due to lack of effect, or variability and small sample size. This result supports the DE gene identifications from RNA-seq.

### 3.4 Network analysis of HTATIP2 depletion

To obtain a more robust picture of the effect of HTATIP2 knock-down on the transcriptome, we repeated our CD4^+^ cell experiment in three independent donors. Each donor’s cells were transfected with either a non-targeting siRNA or an siRNA pool to HTATIP2. After RNA-seq, a differential gene expression analysis was performed in edgeR using the generalized linear model (GLM) functionality to treat each control/knock-down individual as a paired sample. The differentially expressed genes were then submitted to the “core” analysis module of Ingenuity Pathways Analysis to elaborate a gene network.

**Figure 5:**
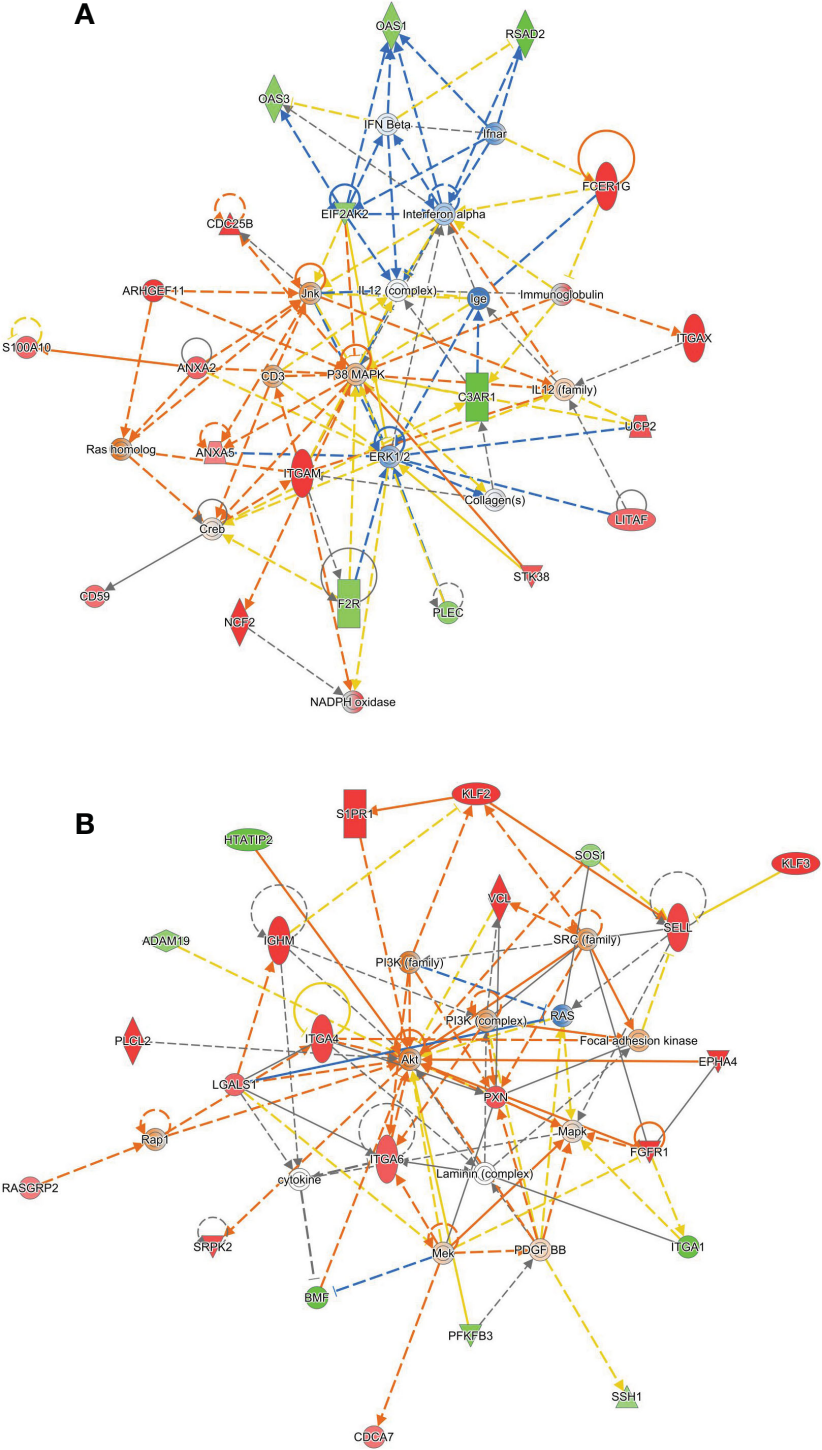
Ingenuity Pathways Analysis identifies two gene networks altered in HTATIP2 knockdown. Primary CD4^+^ T cells were obtained from three donors and transfected with non-targeting siRNA or an siRNA pool against HTATIP2. A paired differential gene expression analysis was used to identify changes in expression. **(A)** A network centered on a hub of MAP kinase signaling. Genes shown in green are downregulated; gene shown in red are upregulated. Solid lines are direct connections; dashed lines are indirect connections. **(B)** A network centered on PI3 kinase/AKT and cell adhesion/migration.

Two networks gave the highest enrichments. The first centers on a hub of MAP kinase signaling, including p38 and JNK (**Figure 5A**). We consulted the UniProt and Entrez Gene summaries through Ingenuity to gain insight into the function of the identified genes. This network showed downregulation of several 2′,5′-oligoadenylate synthetase (OAS) family isoforms, as well as RSAD2 and EIF2AK2, which are antiviral interferon-inducible genes. The α-integrin paralogs ITGAM and ITGAX were upregulated. These are leukocyte-specific adhesion molecules which recognize complement C3, and this is accompanied by downregulation of the G protein-coupled C3 complement receptor (C3AR1) and upregulation of the complement inhibitor CD59 in our experiment. At the same time, these α-integrins play a role in blood clotting, and we observe downregulation of thrombin receptor (F2R). Other MAPK-related proteins include kinase STK38, fibroblast growth factor receptor 1 (FGFR1), and Fc epsilon receptor gamma chain (FCER1G). The structure of this network suggests that HTATIP2 expression promotes antiviral interferon signaling. Viral infection, and the immune responses to it, are thought to be a trigger of β cell autoimmunity (34).

The second network centers on a hub of phosphoinositide-3-kinase and AKT signaling that affects cell motility, adhesion, and chemotaxis (**Figure 5B**). Matrix adhesion-related α-integrin genes are upregulated, including ITGA4, ITGA2, and downregulation of ITGA1. The adhesion- and migration-related tyrosine kinase receptor EPHA4 is upregulated. The cytoskeleton is one downstream effector of this pathway, in which we observe upregulation of paxillin (PXN) and vinculin (VCL), and downregulation of plectin (PLEC). The regulator of actin cytoskeletal dynamics, slingshot protein phosphatase 1 (SSH1), was downregulated. Leukocyte adhesion and infiltration mediator L-selectin (SELL) is highly upregulated. This gene network shows that HTATIP2 expression could enhance β cell autoimmunity by facilitating the infiltration of immune cells into the pancreas from the surrounding microvasculature and migration of these cells into the islets. Enhanced infiltration of immune cells in both exocrine and endocrine pancreas is seen in T1D (35).

Altered gene expression in these signaling pathways, MAPK and PI3K, suggests that the function of T cells might be misdirected or hyperactivated by perturbations to HTATIP2 expression, as might be caused by transcription factors binding to rs10833518.

### 3.5 Effect of HTATIP2 on T-helper cell function

**Figure 6:**
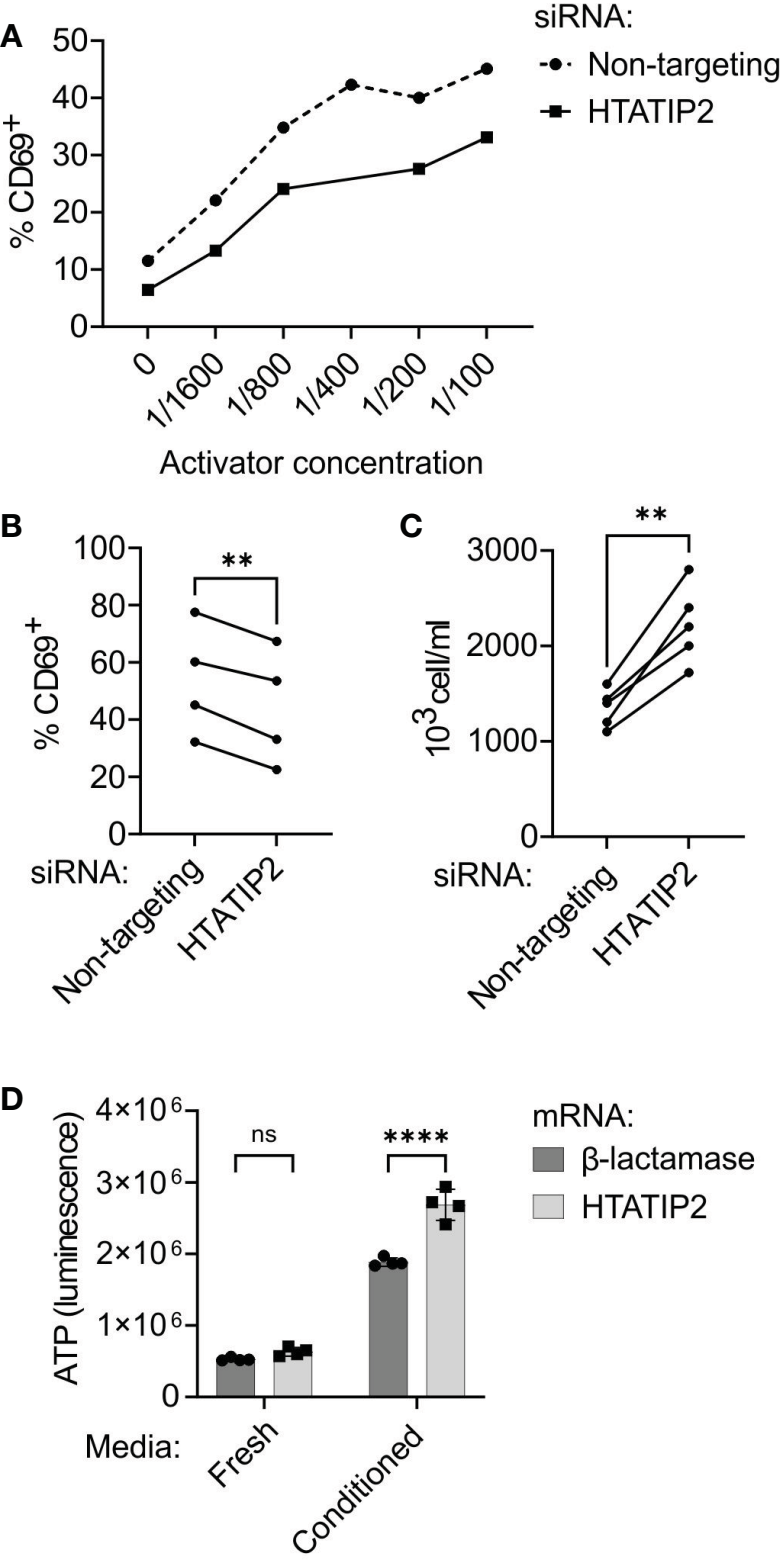
Knock-down or overexpression of HTATIP2 modifies T cell receptor signaling, proliferation, and viability. **(A)** siRNA-transfected CD4^+^ cells were activated with different concentrations of antibodies to T cell receptor CD3/CD28 chains and stained for CD69 expression (representative experiment). The percentage of CD69-positive cells at each concentration is shown for HTATIP2 knock-down and non-targeting control. **(B)** Summary of results from four trials with unique donors of the experiment in panel **(A)**. Activator concentration is 1:100. **(C)** CD4^+^ cells from five donors were transfected with siRNA and seeded at 600,000 cells/ml. The cells were counted 72 h post-transfection. **(D)** CD4^+^ cells from a human donor were transfected with *in-vitro* transcribed mRNA for the control *E coli* β-lactamase gene or for HTATIP2. After electroporation, the cells were seeded in either fresh complete RPMI medium, or 50% spent medium from activated T cells. The cells underwent bioluminescent ATP assay at 72 h post-transfection. **P < 0.01, ****P < 0.0001. ns, non-significant.

We performed HTATIP2 expression modulation in primary human CD4^+^ cells to determine if knock-down or overexpression of HTATIP2 could affect T cell activation, proliferation, or signaling. Primary human CD4^+^ T cells were stimulated for 24 h by anti-CD3/CD28 antibody complexes in the presence or absence of siRNA to HTATIP2. Knock-down of HTATIP2 inhibited T cell activation as assessed by expression of the early activation cell-surface marker CD69 in a dose-dependent manner (**Figure 6A**). This result was replicated with three additional donors (*P* < 0.01, paired *t* test) (**Figure 6B**). Although CD69 expression varies between donors, the amount of TCR inhibition produced by HTATIP2 knock-down is similar.

A consistent observation in the literature on HTATIP2 is that reducing its expression enhances cell proliferation. HTATIP2 knockout mice are prone to spontaneous hepatocellular carcinoma and other tumors (36). We found this to be true of the siRNA-treated primary T cells, with the knock-down cells proliferating ~1.5× greater at 72 h than control cells in five donors in the absence of further stimulation (*P* < 0.01, paired *t* test) (**Figure 6C**). Alterations in cell proliferation and viability are not unexpected in light of changes in gene expression in the MAPK and PI3K pathways, and could be complemented by a reduction in apoptosis (37).

The results of our mRNA-seq experiment suggested that if the A variant of rs10833518 increases HTATIP2 expression in T cells, we might see heightened activation. To test this hypothesis, we overexpressed HTATIP2 by transfection with capped-and-tailed mRNA and assessed viability/proliferation. There was no difference in viability at 72 h when the cells were cultured in fresh medium without stimulation. However, when cells were cultured in filtered spent media from activated T cells, containing secreted cytokines and growth factors, there was a great enhancement in cell proliferation for both control and HTATIP2-overexpressing cells, accompanied by a 50% greater abundance of overexpressing cells (**Figure 6D**). However, we did not observe a difference in CD69 expression when overexpressing HTATIP2 (not shown). These results suggest that increased expression of HTATIP2 can enhance signaling through cytokine receptors and the TCR, leading to increased proliferation and viability. Reduced HTATIP2 only improves proliferation in the absence of TCR activation.

## 4 Discussion

We present results from the first GWAS treating the age of onset and diagnosis of T1D as a unique phenotype in a quantitative trait setting. Two loci are genome-wide significant, one of which is the well-known *MHC* locus, with the other being an association to the *NELL1* gene on chromosome 11, a locus that has not been observed in previous case/control GWAS for T1D or other autoimmune diseases (38). The difference in the age of onset is on-average one year earlier for homozygous carriers of the A risk allele of the putative causal SNP rs10833518. As is almost always the case in complex traits, this SNP may play an important role in some individuals, though the impact is relatively small and the variability is high when averaged across thousands of genetic and environmental backgrounds in the population. In any GWAS study, the statistical power to reject the null hypothesis at genome-wide significance (*P* < 5 × 10^−8^) requires very large samples sizes. The largest GWAS to date considered the trait of adult height in 5.4 million subjects and found over 7000 regions (39) owing to the sample size, as well as the very high heritability of height. The age at diagnosis of T1D is a less genetically-determined trait and clearly must involve environmental factors or triggers such as viral infection, the microbiome, and endocrine hormones. Due to this limited heritability, combined with our modest sample size, we were only able to reject the null hypothesis for two loci, though there are other SNPs with greater-than-chance association in the Q-Q plot. We anticipate that a larger set of age-of-onset data would yield additional loci overlapping the known case/control genes, and that larger case/control studies would identify the chromosome 11 locus.

We nominate HTATIP2, also known as TIP30, as the causal gene for the chromosome 11 association. No other protein-coding gene within 1.5 MB of rs10833518 is expressed in cells of the immune system or has a noticeable impact on T cell gene expression. Our RNA interference experiments revealed that kinases, receptors, and transcription factors in multiple signaling pathways were downregulated by HTATIP2 inhibition, including MAP kinase, PI3-kinase, NFAT, and NF-κB. Based on a gene-network analysis, these signaling perturbations affect the expression of genes involved in antiviral/interferon responses and T cell cytoskeleton, motility, and adhesion. Finally, modulation of the expression level of HTATIP2 shows that higher expression is correlated with increased proliferation and activation marker expression in response to signals through the TCR and cytokine receptors.

Future work must address the molecular mechanism by which HTATIP2 causes these effects. Since the protein was first described (40), several groups have performed affinity purification-mass spectrometry (AP-MS) experiments and found diverse interacting partners (40–44). Furthermore, analysis of the HTATIP2 knockout mouse led to the recognition of its role as a tumor suppressor gene. Consequently, studies have been performed in the areas of signal transduction, endosomal trafficking, cell proliferation, apoptosis, metastasis, protein translation, and metabolism. HTATIP2 expression is frequently down-regulated in cancers (36, 45), which is in agreement with our result that knock-down increases T cell proliferation in the absence of exogenous growth signals. HTATIP2 affects signaling by the epidermal growth factor receptor by regulating endocytosis and EGFR signaling from endosomes (43, 46, 47). Another facet of HTATIP2’s role in subcellular trafficking is in transport of proteins between the nucleus and cytoplasm by interaction with the nuclear pore complex (41, 48). Other studies have shown that when HTATIP2 translocates to the nucleus, it acts as a transcriptional coactivator at gene promoters through interaction with transcription factors such as estrogen receptor coactivator (CIA) and the HIV TAT protein (40, 42). HTATIP2 is a tumor suppressor gene in malignancies such as non-small cell lung cancer and hepatocellular carcinoma because, when active, it induces apoptosis (49, 50), limits metabolic adaption of cancers glucose limitation (51), and inhibits metastasis (52, 53). While our study adds new insights into the role of HTATIP2, further work using a fully *in-vivo* mouse model could help unveil the molecular mechanisms by which HTATIP2 contributes to the pathogenesis of diabetes and autoimmunity.

## Data availability statement

The RNA-seq datasets generated for this study can be found in the NCBI Sequence Read Archive accession PRJNA903865.

## Author contributions

CC performed the experiments and analyzed the data. XC and H-QQ performed the GWAS analysis. ZW analyzed RNA-seq data. JB performed Bayesian fine-mapping. CP provided replication cohort data. HH conceived and supervised the study and obtained funding. All authors contributed to the article and approved the submitted version.
