# Supplementary material for: Genome-wide association study of the age of onset of type 1 diabetes reveals HTATIP2 as a novel T cell regulator
Source: Front Immunol. 2023 Feb 1;14:1101488. doi: 10.3389/fimmu.2023.1101488 (PMC9930890; doi:10.3389/fimmu.2023.1101488)
Supplement: Supplementary file 1 [file DataSheet_1.pdf]

Online Supplement

Genome-wide association study of the age of onset of type 1 diabetes reveals HTATIP2 as a novel T cell regulator

Christopher J. Cardinale[1†], Xiao Chang[1†], Zhi Wei[2], Hui-Qi Qu[1], Jonathan P. Bradfield[3], Constantin Polychronakos[4], and Hakon Hakonarson[1,5*]

[1] Center for Applied Genomics, Children's Hospital of Philadelphia, Philadelphia, Pa., United States

[2] Department of Computer Science, New Jersey Institute of Technology, Newark, N.J., United States

[3] Quantinuum Research LLC, Wayne, Pa., United States

[4] Department of Pediatrics, McGill University, Montreal, Quebec, Canada

[5] Department of Pediatrics, Perelman School of Medicine at the University of Pennsylvania, Philadelphia, Pa., United States

† These authors contributed equally.

* **Correspondence:**

Hakon Hakonarson, MD, PhD

hakonarson@chop.edu

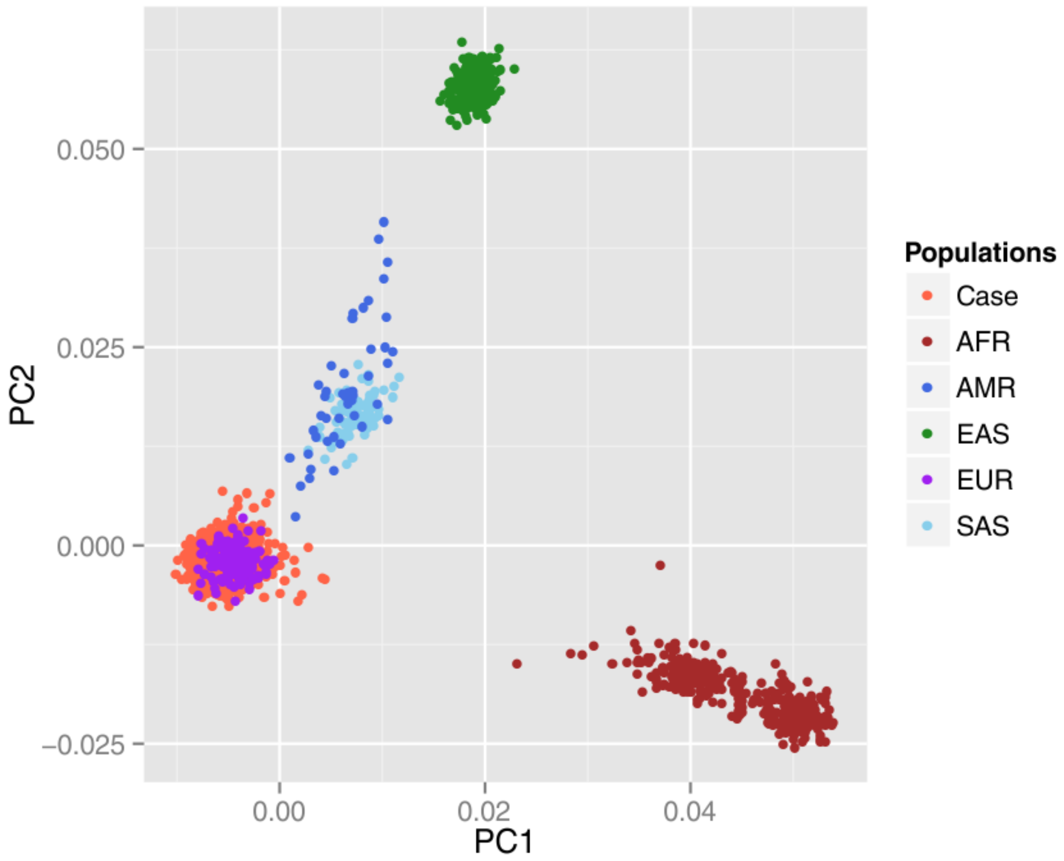

,

Supplementary Figure S1. Principal components analysis of the discovery cohort. Case subjects are within the European population. Other 1000 Genomes Project populations are shown. Case = type 1 diabetes subjects. AFR = African. AMR = Native American. EAS = East Asian. EUR = European. SAS = South Asian.

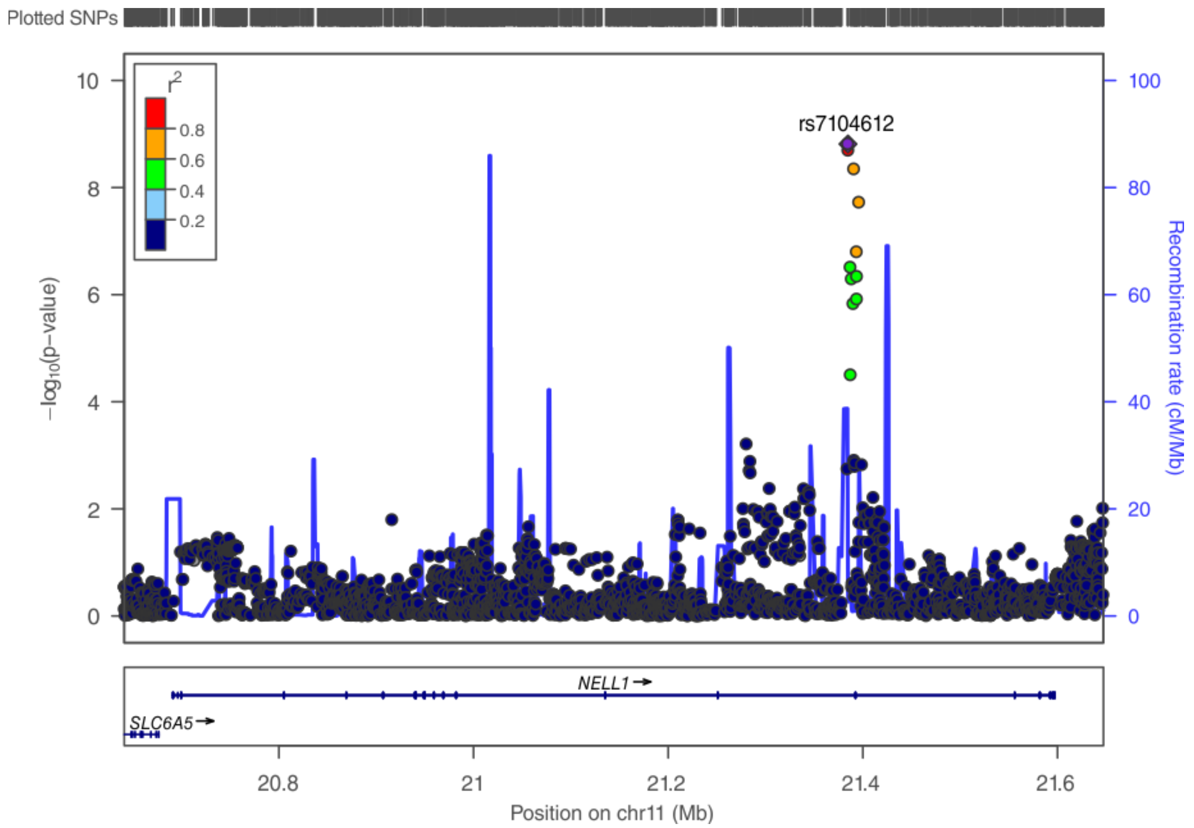

Supplementary Figure S2. Regional association plot of the meta-analysis (generated in LocusZoom) identifying the lead SNP rs7104612 within a haplotype block containing exon 14 of the *NELL1* gene. SNPs in linkage disequilibrium with the lead SNP are color-coded by the r^2 of the LD.

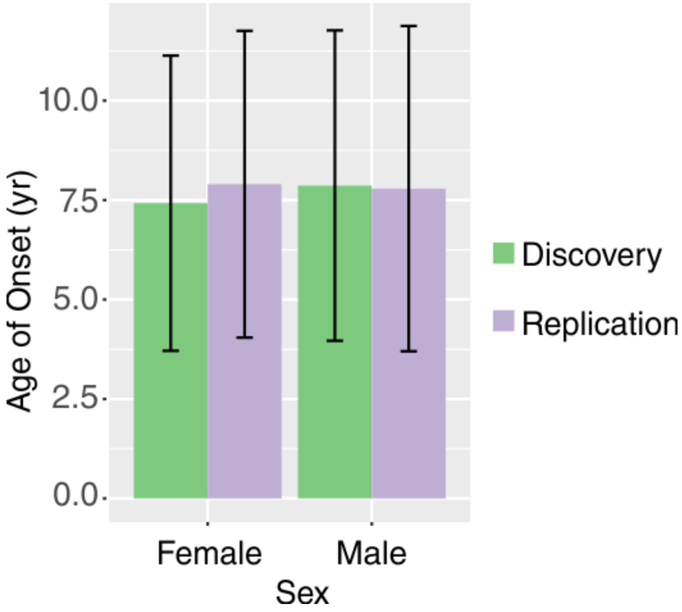

Supplementary Figure S3. The age of onset for subjects in the discovery and replication cohorts by sex (average with standard deviation). There are no statistically significant differences.

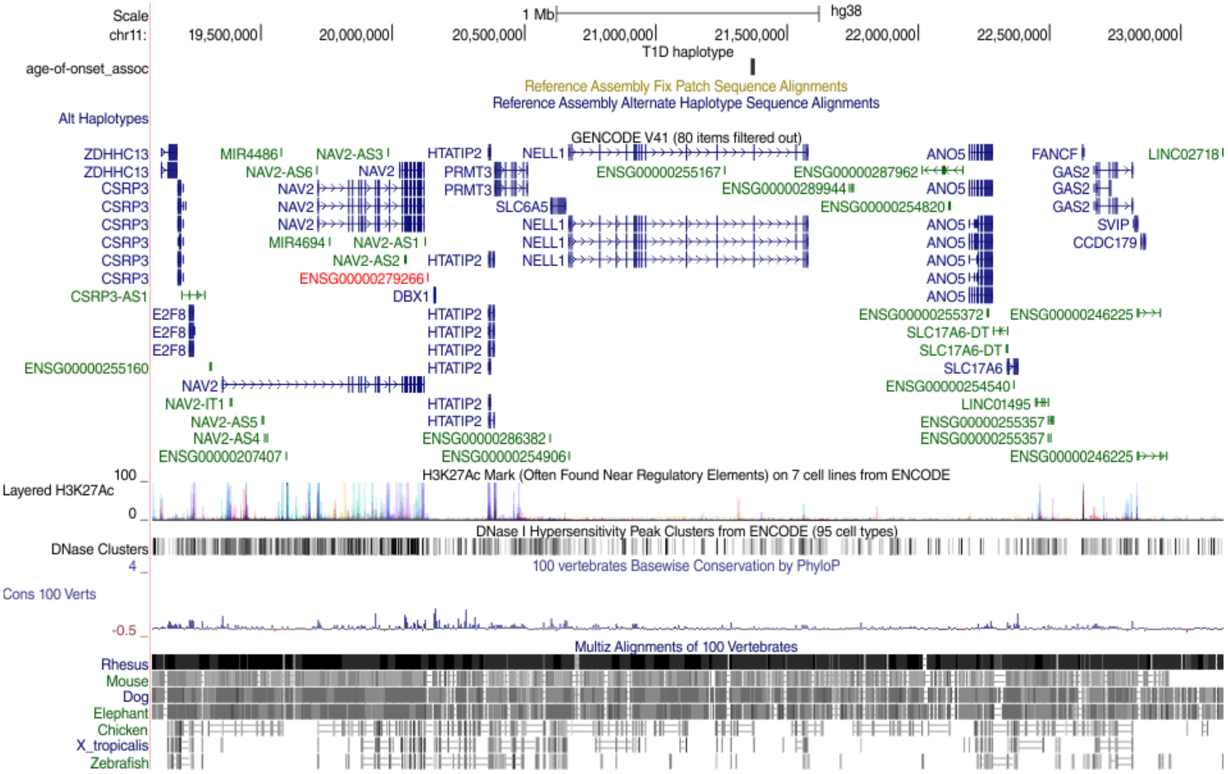

Supplementary Figure S4. Map of the *NELL1* locus generated in UCSC Genome Browser. This 4-megabase region is centered on the *NELL1* gene. The T1D haplotype track shows the location of the associated SNPs. The second track shows Gencode v 41 protein- and RNA-coding genes. The bottom tracks are for H3K27 acetylation ChIP-seq, DNase hypersensitive sites, and evolutionary conservation.

siRNA Sequences

Non-targeting	AUG UAU UGG CCU GUA UUA G
HTATIP2	GGG UAC CAC CAG AGG GAA A<br>CUU UCA AGG UCA UGA UGU U<br>GCA GAA UAA AUC CGU CUU U<br>UCG ACG AGG AAG CUU AUA A
PRMT3	GAA GAA AGC AGU UAU UCC A<br>GCA UAU AGA UUG CCA UAC G<br>CAG CAU GGU UCA UAA ACA U<br>CCC AAU GGA CUC AGU GAA A
FANCF	GGG AGG AGU UGC ACA AUA G<br>GGU CAA CGU UUG CAC UAU G<br>UAA CUG CCC UGG AGA CCU G<br>CUU AUU AGC UCU UCG UAG U
SVIP	CCU UAA GUG CAA UG CUA A<br>GUA AGG AUC UUU GUA GUA A<br>ACU UAU UAG AGA ACG AUC A<br>CAA GUG GGC UUC AUC AAU U

PCR Primers

HTATIP2 qPCR F	ATG ACT ACG CCT CTG CCT TTC A
HTATIP2 qPCR R	TCG GTC AAC ACG AAC AAA TCC C
MAP3K2 RT fwd	CAG ATT CTG GAG GGT GTC CAT TAT TTG
MAP3K2 RT rev	CCT AGT TTG ACG TTG CCT GTT GAA TCT
IL6ST RT fwd	ACA GAT GAA GGT GGG AAG GAT GGT C
IL6ST RT rev	GGT GAC CAC TGG GCA ATA TGA CTC TT
RORA RT fwd	ATC ATC AGG AAT CCA TTA TGG TGT CAT T
RORA RT rev	ACG AGG ACA GGA GTA GGT GGC ATT
RICTOR RT fwd	CGA GCA TGC ATT GCC ATT ATC TGT
RICTOR RT rev	TGG CAA TCG ATC ACA TTT TTC AAG A
IKZF2 RT fwd	AGC CGT TCA AAT GTC CTT TCT GTA GC
IKZF2 RT rev	CGT TCC TTG TGC TCC TCC AGT GA
REL RT fwd	AAA GAC TGC AGA GAC GGC TAC TAT GA
REL RT rev	GAT TGA TTC CTG CCT TTA TTC TTG TA

ELK4 RT fwd	TGG CTC GTC TCT GGG GGA TTC
ELK4 RT rev	CCC TCA ATC CTG CCC ACT GTC A
PIK3C2A RT fwd	ATG AGG AGA TGG CAG CTT TTT GTC
PIK3C2A RT rev	ATG TTT CTT TGT GCT GTG ACT GGA CTT A
NFAT5 ver 2 F	CAA TGA GTC AGA CAA GCG GTG GTG
NFAT5 ver 2 R	ATG GTA GGG CTG GAA GAG GTG GTA A
